# Risk factors for low birth weight in hospitals of North Wello zone, Ethiopia: A case-control study

**DOI:** 10.1371/journal.pone.0213054

**Published:** 2019-03-20

**Authors:** Tesfahun Mulatu Wachamo, Nigus Bililign Yimer, Asmamaw Demis Bizuneh

**Affiliations:** 1 Department of Public Health, Faculty of Health Sciences, Woldia University, Woldia, Ethiopia; 2 Department of Midwifery, Faculty of Health Sciences, Woldia University, Woldia, Ethiopia; 3 Department of Nursing, Faculty of Health Sciences, Woldia University, Woldia, Ethiopia; National Institute of Public Health, MEXICO

## Abstract

**Background:**

Low birth weight at birth is an important underlying contributor for neonatal and infant mortality. It accounts for nearly half of all perinatal deaths. Identifying predictors of low birth weight is the first essential step in designing appropriate management strategies. Hence, this study aimed to identify risk factors for low birth weight in hospitals of northeastern Ethiopia.

**Methods:**

An institution based case-control study design was conducted from 10^th^ April to 15^th^ December 2016. Three hundred sixty mother-infant pairs (120 low birth weight babies as cases and 240 normal birth weights as controls) were included in the study. Data were collected by face-to-face interview. Univariable and multivariable logistic regression models were computed to examine the effect of independent variables on outcome variable using SPSS 20.0. Variables with p-value <0.05 were considered statistically significant.

**Results:**

The mean (±SD) gestational age and birth weight (±SD) were 39.2 (±1.38) weeks and 2800 (±612), grams respectively. Partner’s education/being illiterate (AOR: 4.09; 95% CI 1.45, 11.50), antenatal care visit at private health institutions (AOR: 0.13; 95% CI 0.02, 0.66), having history of obstetric complications (AOR: 5.70; 95% CI 2.38, 13.63), maternal weight during pregnancy (AOR: 4.04; 95% CI 1.50, 10.84) and gravidity (AOR: 0.36; 95% CI 0.18, 0.73) were significantly associated with low birth weight. Additionally, a site for water storage and water treatment were significant environmental factors.

**Conclusion:**

Maternal weight during pregnancy, paternal education, previous obstetric complication and place of antenatal follow-up were associated with low birth weight. The risk factors identified in this study are preventable. Thus, nutritional counseling, health education on improvement of lifestyle and early recognition and treatment of complications are the recommended interventions.

## Introduction

Low birth weight (LBW) is defined as a birth weight less than 2500g and LBW infants are at greater risk of death and disability [1]. Globally, 2.6 million newborns died in 2016. Half of these deaths occurred in India, Pakistan, Nigeria, the Democratic Republic of the Congo and Ethiopia. The most common causes of these deaths were birth asphyxia, infection, complications of preterm birth and birth defects in the early neonatal period [2]. Thirty percent of deaths were attributed to premature birth and low birth weight[3]. According to the recent report of the United Nations Children's Fund (UNICEF), the neonatal mortality rate of Ethiopia was 28 per 1000 live births in 2016 [4].

Globally, an estimated 15% to 20% of all births are LBW [5, 6]. The prevalence of low birth weight in Senegal, Burkina Faso, Malawi, Ghana, and Uganda was, respectively, 15.7%, 13.4%, 12.1%, 10.2%, and 10% [7]. Currently, thirteen percent of Ethiopian babies are low birth weight [8]. Studies in Ethiopia reported 8.8% and 10.5% prevalence of low birth weight in Addis Ababa and Tigray region, respectively [9, 10].

Very low birth weight infants have decreased cognitive, language and motor functions [11]. A systematic review of low birth weight in Africa showed an increased risk of death, growth retardation and delayed neurodevelopment among very low birth weight and extremely low birth weight children [12]. Furthermore, newborns with low birth weight were at higher risk of stillbirth, low Apgar score, admission to neonatal intensive care unit and early neonatal death in Zambia [13]. Additionally, low birth weight was associated with a risk of hypertension later in life [14].

One cohort study showed vomiting during the early trimester of pregnancy was associated with a higher risk of low birth weight [15]. In developing countries, maternal age, illiteracy, antenatal care follow-up, body mass index and socioeconomic status were predictors of low birth weight [16]. Antepartum hemorrhage, hypertensive disorders of pregnancy and primiparity were associated with low birth weight in a Gambian study [17]. Additionally, low birth weight was associated with maternal anemia [18] and malaria during pregnancy [19], intrauterine growth restriction (IUGR) and premature birth [20]. Epidemiological studies conducted in Ethiopia identified some risk factors for low birth weight. Education and occupational status [21], maternal stature and weight [22], hypertensive disorders during pregnancy [23], congenital malformations [24] and indoor pollution from the type of fuel used for cooking [21] were predictors of low birth weight.

In 2014, the World Health Organization (WHO) identified evidence-based interventions to prevent low birth weight [6]. Overview of systematic reviews showed the positive effect of multiple micronutrient supplements and preventive antimalarial drugs during pregnancy on low birth weight [5, 25]. Additionally, intake of Docosahexaenoic Acid (DHA) was associated with lower rates of low birth weight [26]. In Ethiopia, one prospective cohort reported lower risks of adverse pregnancy outcomes (including low birth weight) among consumers of dairy, fruits and dark green leafy vegetables [27].

Ethiopia has made a notable achievement in reducing neonatal mortality during the era of the Millennium Development Goals (MDGs) [28]. Goal 3 of the new Sustainable Development Goals (SDGs) targets broader health topics related to newborn and child health. Because SDGs address a range of socio-economic and environmental risk factors for health-related problems [29], identifying risk factors for low birth weight has many benefits to set preventive and treatment strategies. The Ethiopian government aspires to decrease neonatal mortality to 10 per 1000 live births by 2035 [30]. The findings of this study will add evidence for intervention designers to establish appropriate management protocols, targeted to the study area. The study area is located in a region where neonatal mortality and stunting are high in the country [8]. Therefore, this study aimed to identify risk factors for low birth weight in hospitals of North Wello Zone, Ethiopia.

## Methods and materials

### Study design and setting

A hospital-based unmatched case-control study design was employed from April 10 to December 15, 2016, in North Wello zone hospitals. This zone has an estimated total population of 1,500,303 (752,895 men and 747,408 women). Based on reports from the zone’s health sector office, North Wello zone has 3 hospitals and 64 functional health centers. The study was conducted in the three hospitals; Woldia General Hospital, Lalibela hospital, and Kobo hospital, which are located 520, 700 and 571 kilometers away from Addis Ababa respectively. In 2015, there were 6,013 births in Woldia, Lalibela and Kobo towns.

### Study population

Newborns who were born in the three public hospitals during the study period (8 months) were the source population of this study. Mothers of the newborns were the source of the information. Live newborns delivered at term without known risk factors (i.e. intrauterine growth restriction) of low birth weight were included in the study. Mothers who gave birth before 37 completed weeks of gestation and mothers with medical conditions, which affect birth weight (i.e. hypertensive disorders of pregnancy, diabetes mellitus), were excluded from the study. Mothers who gave birth to neonates weighing less than 2500 grams were cases and neonates ≥2500 grams were controls.

### Study variables

The outcome/dependent variable was low birth weight. The exposure/independent variables were socio-demographic variables (maternal age, education, occupation,), obstetric factors (gravidity, parity, ANC visit, place of delivery, place of prenatal visit, information about danger signs, complications during last birth, maternal weight and height and gestational age at first booking) and environmental characteristics (water treatment, site of deification, Khat chewing, source and storage of drinking water, handwashing, presence of windows, separate room for kitchen and family water consumption).

### Sample size determination

The sample size was determined using the proportion difference approach with the following assumptions: 95% confidence level (Zα/_2_ = 1.96), power (Zβ = 0.84), control to case ratio 2:1 (r = 2), odds ratio to be detected 2.1 and 20% [21] of control group to be exposed. Adding a 5% non-response rate, the final required sample size was 375 (125 cases and 250 controls).

### Sampling and data collection procedure

All the three hospitals in the zone were selected purposively. Babies of mothers who delivered during the study periods were measured using a calibrated scale within 15 minutes of delivery. Cases (birth weight less than 2500 grams) were included in the study and two consecutive mothers in the controls (birth weight between 2500 grams and 4000 grams) were interviewed.

Data were collected using a pre-tested and structured questionnaire through a face-to-face interview. The tool was adapted from previously published work [21]. The questionnaire was composed of three sections: socio-demographic characteristics, maternal/obstetric characteristics, and environmental characteristics. First, it was prepared in English, translated to Amharic and back-translated by fluent speakers of both languages. A maternal health expert checked the validity of the tool and pilot study was conducted on 5% of the sample size in Alamata hospital. The Amharic version was used to collect the data. Six diploma-qualified midwives collected the data (one day and one night at each hospital). Three Bachelor of Science (BSc) qualified midwives supervised data collection and checked the completeness of the questionnaire. The principal investigator trained the supervisors and data collectors about the tool and procedure of data collection. Supervisors checked the completeness of the data.

### Operational/term definitions

**Low birth weight (LBW)**: In this study, a newborn was considered as low birth weight if they weighed less than 2500 grams.

**Normal birth weight (NBW)**: birth weight between 2500 and 4000 grams.

**Preterm birth**: delivery before 37 completed weeks of gestation.

### Statistical analysis

After checking for completeness, the data were coded and entered into a statistical package for social sciences (SPSS) version 20.0-computer software for analysis. Frequency distributions including cross tabulation between cases and controls were completed. Univariable and multivariable logistic regression analyses were computed to see the effect of independent variables on the outcome variable.

In the univariable logistic regression analysis, the variables entered were husband education, income, maternal occupation and education, place of ANC visit, maternal age, height, weight, information about obstetric danger signs, gravidity, history of obstetric complications, gestational age at first antenatal care visit, source of drinking water, water treatment before drinking presence of windows, separate room for kitchen, khat chewing, site of storage for water, family water consumption, hand washing, solid waste disposal and latrine type.

Variables with p-value ≤0.2 in the univariable analysis (husband’s education, place of ANC visit, informed about danger signs during ANC visit, history of obstetric complications, maternal weight, height, age, gravidity, gestational age at first antenatal booking, khat chewing, source of drinking water, site for storage of water, water treatment, hand washing, separate room for cooking, family water consumption and presence of windows) were entered to multivariable logistic regression analysis. Backward stepwise logistic regression method was used. Hosmer and Lemeshow goodness-of-fit was used to test model fitness. Statistically significant variables were declared at p-value less than 0.05. Odds ratio (OR) with 95% confidence interval was used to describe the strength of association between the independent variables and outcome variable. A brief sensitivity analysis was done for the model (S1 Table).

### Ethical approval and consent to participate

The institutional review board (IRB) of Woldia University approved this study. A letter of permission was secured from North Wello zone health sector. Informed verbal consent was obtained from study participants after explaining the objectives of the study. The tool had attached consent form. Assent was received from participants less than 18 years old and permission was obtained from primary guardians available during data collection. As most of our study participants unable to read and write, the data collectors document consent by circling ‘yes’ or ‘No’ options provided at the end of the form. Confidentiality was ensured by anonymizing the respondents' name.

## Results

### Socio-demographic characteristics of the respondents

A total of 360 cases and controls were included in the study, yielding a response rate of 96%. The mean age (±SD) of the respondents was 27.7 (±5.6) which ranged from 15 to 45 years. About three-quarter of the cases and 83% of the controls were between the age group of 21 to 35. Most of the respondents practiced Ethiopian Orthodox Christianity (more than three-fourths from cases and 70.8% from controls). Majority of the respondents had no formal education and were housewives [Table 1].

**Table 1:** Socio-demographic characteristics of mothers in North Wello zone Hospitals, Ethiopia, December 2016.

Variables	Low birth weight		Normal birth weight	
N	%	N	%
Mothers age (n = 360)				
≤ 20	19	15.8	15	6.2
21–35	89	74.2	200	83.3
>35	12	10.0	25	10.4
Marital status (n = 360)				
Married	97	80.8	208	86.7
Divorced	12	10.0	9	3.8
Separated	2	1.7	6	2.5
Single	8	6.7	12	5
Widowed	1	0.8	5	2.1
Religion (n = 360)				
Ethiopian Orthodox Christianity	92	76.7	170	70.8
Protestant	5	4.2	19	7.9
Muslim	23	19.2	51	21.2
Residence				
Urban	70	58.3	147	61.2
Rural	50	41.7	93	38.8
Ethnicity (n = 360)				
Amhara	107	89.2	197	82.1
Tigre	13	10.8	43	17.9
Maternal formal education (n = 360)				
Yes	87	72.5	164	68.3
No	33	27.5	76	31.7
Education status of the mother (n = 251)				
Primary	19	21.8	46	28.0
Secondary	34	39.1	55	33.5
More than secondary	34	39.1	63	38.4
Educational status of the husband (n = 305)				
Illiterate	18	18.6	24	11.5
Read and write only	31	32	53	25.5
Primary	12	12.4	40	19.2
Secondary and above	36	37.1	91	43.8
Maternal occupation (n = 360)				
Housewife	49	40.8	109	45.4
Farmer	26	21.7	26	10.8
Private employee	4	3.3	19	7.9
Merchant	13	10.8	43	17.9
Daily laborer	10	8.3	13	5.4
Gov’t employee	18	15.0	26	10.8
Others*			4	1.7
Husband occupation (n = 305)				
Farmer	39	40.6	65	31.1
Merchant	18	18.8	56	26.8
Private employee	7	7.3	29	13.9
Daily laborer	3	3.1	5	2.4
Gov’t employee	29	30.2	54	25.8
Monthly income of the family (ETB) (n = 360)				
< 500	55	45.8	107	44.6
500–1500	22	18.3	36	15.0
> 1500	43	35.8	97	40.4

*student; ETB Ethiopian Birr

### Obstetric characteristics

The mean gestational age (±SD) and birth weight (±SD) were 39.2 (±1.38) weeks and 2800 (±612) grams respectively. The mean age of respondents during their first and last birth was 22.2 and 26.1 years, respectively. More than one-third of mothers in the cases and 40.8% of the controls had their first birth before the age of 20. Approximately 23% of mothers in the cases weighed below 50 kilograms during their pregnancy. Approximately one-third of mothers in the cases encountered obstetric complications during pregnancy. Vaginal bleeding was the most frequently mentioned danger sign by 34.8% of cases and 59.7% of controls [Table 2].

**Table 2:** Maternal/Obstetric characteristics of respondents in North Wello zone Hospitals, Ethiopia, December 2016.

Variables	Low birth weight		Normal birth weight	
N	%	N	%
Maternal age at first birth (n = 360)				
≤ 20	46	38.3	98	40.8
> 20	74	61.7	142	59.2
Maternal age at last birth (n = 360)				
< 35	115	95.8	225	93.8
≥ 35	5	4.2	15	6.2
Maternal weight in kilograms (n = 360)				
< 50	28	23.3	20	8.3
≥ 50	92	76.7	220	91.7
Maternal height in meters (360)				
≤ 1.50	17	14.2	16	6.7
> 1.50	103	85.8	224	93.3
Maternal BMI (Kg/m^2^) (n = 360)				
<18.5	8	6.7	4	1.7
18.5–25.0	95	79.2	181	75.4
>25.0	17	14.2	55	22.9
ANC follow up (n = 360)*				
Yes	107	89.2	210	87.5
No	13	10.8	30	12.5
Number of ANC visits (n = 317)				
1	6	5.5	5	2.4
2–4	99	90.8	191	91.8
>4	4	3.7	12	5.8
Place of ANC visit (n = 317)				
Public health facility	106	97.2	175	84.1
Private health facility	3	2.8	33	15.9
Number of pregnancies (n = 360)				
1	54	45.0	67	27.9
2–4	53	44.2	150	62.5
> 4	13	10.8	23	9.6
Gestational age (weeks) at first ANC visit (n = 309)				
≤ 16	32	30.5	88	43.1
17–24	52	49.5	84	41.2
> 24	21	20.0	32	15.7
Having a history of obstetric complications during				
pregnancy (n = 360)				
Yes	35	29.2	29	12.1
No	85	70.8	211	87.9
Having a history of abortion (n = 360)				
Yes	23	19.2	41	17.1
No	97	80.8	199	82.9
danger signs informed for the mothers (n = 360)**				
Vaginal bleeding	88	34.8	151	59.7
Vaginal gush of fluid	60	23.7	104	41.1
Severe headache	81	32.0	132	52.2
Blurred vision	70	27.7	94	37.2
Fever	59	23.3	96	37.9
Abdominal pain	64	25.3	77	30.4
danger signs encountered by the mothers (n = 71)**	8			
Vaginal bleeding	7	12.9	7	11.3
Vaginal gush of fluid	11	11.3	12	19.4
Severe headache	7	17.7	6	9.7
Blurred vision	2	11.3	1	1.6
Fever	8	3.2	0	0
Abdominal pain		12.9	2	3.2

*at least one visit

**variables with multiple responses

### Environmental characteristics and lifestyle of participants

The average daily water consumption per liter per household of cases was 89.2% and the majority (60%) of the cases did not treat water before drinking. Nearly one-fourth of the cases and one-fifth of the controls had no latrine. Most of the respondents (75.8% cases and 54.2% controls) used firewood for cooking. Approximately one-third of women in the case group drink alcohol and 11% chewed Khat [Table 3].

**Table 3:** Environmental and lifestyle characteristics of respondents in North Wello Zone Hospitals, Ethiopia, December 2016.

Variables	Low birth weight		Normal birth weight	
N	%	N	%
Source of drinking water (n = 360)				
Protected	91	75.8	203	84.6
Not protected	29	24.2	37	15.4
Household water consumption per liter/day (n = 360)				
< 50	107	89.2	187	77.9
≥ 50	13	10.8	53	22.1
Average time required to fetch water (n = 360)				
< 30 minutes	112	93.3	213	88.8
≥ 30 minutes	8	6.7	27	11.2
Presence of latrine (n = 360)				
Yes	91	75.8	194	80.8
No	29	24.2	46	19.2
Having a separate room for kitchen (n = 360)				
Yes	72	60.0	174	72.5
No	48	40.0	66	27.5
Type of fuel mainly used for cooking (n = 360)				
Wood	91	75.8	130	54.2
Kerosene	3	2.5	9	3.2
Electricity	18	15.0	85	35.4
Animal Dung	8	6.7	16	6.7
Treating drinking water (n = 360)				
Yes	48	40.0	59	24.6
No	72	60.0	181	75.4
Time of hand washing*				
After visiting latrine	92	25.6	170	47.4
Before preparing food	102	28.4	155	43.2
Before serving food	97	27.0	142	39.6
After eating	95	26.5	192	53.5
After cleaning child feces	80	22.3	169	47.1
Drinking alcohol during pregnancy (n = 360)				
Yes	38	31.7	76	31.7
No	82	68.3	164	68.3
Type of alcohol consumed by the mothers (n = 165)*				
Tela^+^	35	30.4	58	50.4
Tej^+^	16	13.9	14	12.2
Areke^+^	2	1.7	3	2.6
Beer/draft	7	6.1	28	24.3
Wine	2	1.7	0	0
Khat chewing (n = 360)				
Yes	13	10.8	14	5.8
No	107	89.2	226	94.2

*variables with multiple responses

^+^traditional homemade alcohols in Ethiopia

### Risk factors for low birth weight

Husband’s education was found to be significantly associated with low birth weight. Illiterate mothers were 4 times more likely to have low birth weight (AOR: 4.09; 95% CI 1.45, 11.50). Mothers who attended their ANC visit at private health facilities were 87% less likely to have low birth weight infant (AOR: 0.13; 95% CI 0.02, 0.66). Additionally, mothers who did experience any obstetric complications during their last pregnancy had a higher chance of having low birth weight infant (AOR: 5.70; 95% CI 2.38, 13.63).

Maternal weight during pregnancy was another variable that was significantly associated with low birth weight. Mothers who weighed less than 50 kilograms were 4 times (AOR: 4.04; 95% CI 1.50, 10.84) more likely to have low birth weight baby than mothers who weighed more than 50 kilograms. Gravidity (2–4 pregnancies) also showed significant association with low birth weight (AOR: 0.36; 95% CI 0.18, 0.73). From the environmental factors, the site of storage for water and water treatment were statistically significant variables for low birth weight [Table 4].

**Table 4:** Association of socio-demographic, obstetric and environmental risk factors with low birth weight in North Wello Zone Hospitals, Ethiopia, December 2016.

Variables	Birth weight N (%)		COR (95% CI)	AOR (95% CI)
	LBW	NBW		
Husband education				
Illiterate	19 (19.4)	24 (11.5)	2.00 (0.98, 4.09)	4.09 (1.45, 11.50)*
Read and write only	31 (31.6)	53 (25.5)	1.48 (0.82, 2.66)	1.85 (0.81, 4.19)
Primary school	12 (12.2)	40 (19.2)	0.76 (0.34, 1.60)	0.83 (0.32, 2.18)
Secondary and above	36 (36.7)	91 (43.8)	1	1
Place of ANC visit				
Public HI	106 (97.2)	175 (84.1)	1	1
Private HI	3 (2.8)	33 (15.9)	6.66 (1.99, 22.26)	0.13 (0.02, 0.66)*
Informed about danger				
signs of pregnancy				
Yes	91 (82.0)	162 (75.7)	1	1
No	20 (18.0)	52 (24.3)	0.68 (0.38, 1.22)	0.82 (0.31, 2.14)
Complications during				
last birth				
Yes	35 (29.2)	29 (12.1)	2.99 (1.72, 5.20	**5.70 (2.38, 13.63**)**
No	85 (70.8)	211 (87.9)	1	1
Maternal weight				
<50 kg	28 (23.3)	20 (8.3)	3.35 (1.79, 6.24)	4.04 (1.50, 10.84)*
≥50 kg	92 (76.7)	211 (91.7)	1	1
Maternal height				
≤1.5 meters	17 (14.2)	16 (6.7)	2.31 (1.12, 4.75)	0.46 (0.14, 1.50)
>1.5 meters	103 (85.8)	224 (93.3)	1	1
Maternal age				
≤20	19 (15.8)	15 (6.2)	2.64 (1.005, 6.93)	1.46 (0.14, 14.60)
21–35	89 (74.2)	200 (83.3)	0.927 (0.446, 1.92)	2.48 (0.26, 23.64)
>35	12 (10.0)	25 (10.4)	1	1
Gravidity				
1	54 (45.0)	67 (27.9)	1	1
2–4	53 (44.2)	150 (62.5)	0.44 (0.27, 0.70)	0.36 (0.18, 0.73)*
>4	13 (10.8)	23 (9.6)	0.70 (0.32, 1.51)	0.44 (0.15, 1.31)
Gestational age at first				
ANC visit				
≤16	32 (30.5)	88 (43.1)	1	1
17–24	52 (49.5)	84 (41.2)	1.70 (1.00, 2.89)	1.16 (0.53, 2.52)
>24	21 (20.0)	32 (15.7)	1.80 (0.91, 3.57)	2.10 (0.77, 5.70)
Khat chewing				
Yes	13 (10.8)	14 (5.2)	1.96 (0.89, 4.32)	1.42 (0.34, 5.61)
No	107 (89.2)	226 (94.2)	1	1
Source of drinking water				
Protected	91 (75.8)	203 (84.6)	1	1
Not protected	29 (24.2)	37 (15.4)	1.42 (0.78, 2.55)	0.56 (0.18, 1.72)
Site of storage for				
drinking water				
Closed container	87 (72.5)	148 (61.7)	1	1
Open container	33 (27.5)	92 (38.3)	0.61 (0.38, 0.98)	0.25 (0.12, 0.50)**
Water treatment				
Yes	48 (40.0)	59 (24.6)	1	1
No	72 (60.0)	181 (75.4)	0.48 (0.30, 0.78)	0.27 (0.13, 0.56)**
Hand washing				
Water only	26 (21.7)	67 (27.9)	0.72 (0.43, 1.12)	0.53 (0.21, 1.34)
Soap and water	94 (78.3)	173 (72.1)	1	1
Presence of windows				
Yes	93 (77.5)	212 (88.3)	1	1
No	27 (22.5)	28 (11.7)	2.12 (1.23, 3.93)	1.20 (0.34, 4.28)
Separate room for				
kitchen				
Yes	72 (60.0)	174 (72.5)	1	1
No	48 (40.0)	66 (27.5)	1.76 (1.10, 2.79)	1.74 (0.67, 4.54)
Family water				
consumption/L/day				
<50	107 (89.2)	187 (77.9)	2.38 (1.24, 4.57)	1.23 (0.47, 3.24)
≥50	13 (10.8)	53 (22.1)	1	1

COR crude odds ratio; AOR adjusted odds ratio; CI confidence interval; HI health institution

*statistically significant at p-value<0.05

**p-value less than 0.001; Hosmer and Lemeshow test 0.23

## Discussion

Low birth weight is one of the leading causes of neonatal mortality and is influenced by various socio-economic, maternal and environmental factors [31]. This study identified some socio-economic, obstetric and environmental risk factors for low birth weight in the study area.

This study revealed that husband’s education was associated with low birth weight. A finding from the Ethiopian Demographic and Health Survey (EDHS) showed that husband’s educational status was significantly associated with low birth weight [32]. The 2016 EDHS also reported that births to mothers without education were more likely to be low birth weight [8]. Similarly, maternal illiteracy was associated with low birth weight in one Iranian study [33]. This might be due to the fact that families with formal education could have better nutrition and access to health facilities during pregnancy. Additionally, rural residence increased the odds of low birth weight in a study done in northern Ethiopia and Iran [10, 33]. This suggests that health messages should be tailored based on education level. However, it should be noted that the odds of this variable in this study represent only married women.

Epidemiologic studies in Ethiopia showed that lack of antenatal care was significantly associated with low birth weight [21, 34]. In this study, the place of antenatal care (ANC) follow-up was another significant variable associated with low birth weight. Mothers who attended ANC follow-ups at private health institutions were less likely to have low birth weight babies. A study conducted among women attending private hospitals in Pakistan showed that socio-economic factors (education, income) were not associated with low birth weight [35]. Additionally, women in higher wealth quantile with some education had lower odds of low birth weight babies [36]. This could be due to the indirect effect of income, which favors better access to nutrition and other basic needs during pregnancy. Additionally, appropriate service care provided by the private sector could explain this [37]. Furthermore, this may suggest the need of universal standard prenatal interventions. This finding may be also alarm for health sector officials to improve the quality of prenatal services in government health institutions.

This study revealed that maternal weight was a predictor of low birth weight. Mothers who weighed less than 50 kilograms during their pregnancy were at higher risk of delivering a baby with low birth weight than mothers weighed more than 50 kilograms. This finding was consistent with studies conducted in Ethiopia and other countries [21, 34, 38, 39]. A review article also demonstrated that optimal weight gain during pregnancy was associated with desired birth weight [40]. Furthermore, maternal BMI was significantly associated with birth weight in Senegal [7]. This indicates the need for promotion of ANC follow-up and nutritional counseling during pregnancy. Adapting and implementing nutritional projects of other countries may be important to prevent low birth weight.

Additionally, number of pregnancies was an obstetric factor associated with low birth weight in the present study. Mothers who were in their second to fourth pregnancy were less likely to have low birth weight infant than primigravidas. Parity was significantly associated with birth weight in northwest Ethiopia and the Gambia and London [17, 22] [33]. Another hospital based study in London [38] showed association of parity with low birth weight. A cross-sectional study conducted in Central Africa revealed that adolescent women had significant risks of delivering a low birth weight baby [41]. This might be due to the effect of placental factors as gravidity/parity increases. A systematic review and meta-analysis revealed an association between nulliparity with low birth weight [42]. This shows the need of universal and quality prenatal care, nutritional counseling to all pregnant women. Additionally, early marriage and teenage pregnancy should be discouraged to prevent low birth weight in this group of women.

This study identified history of obstetric complications as a risk factor for low birth weight. Study subjects who did not experience any obstetric complications during their past pregnancies had decreased risk of low birth weight babies. Similar findings were reported by other descriptive studies in Ethiopia [21, 23, 32]. This may be an indication for the importance of early detection and treatment of complications during ANC visits. Moreover, socio-economic characteristics of women should be taken in to account. This finding may also an indication to launch the current WHO recommendations on frequency of ANC visits.

The present finding showed the protective effect of storing water in an open container and drinking without treatment for low birth weight. Studies showed that prenatal exposure to chlorination by-products in drinking water was associated with risks of small for gestational age [43, 44]. A cohort study in India reported that low birth weight was not associated with type and time to a drinking water source [45]. On the other hand, arsenic exposure in drinking water was associated with increased odds of low birth weight [46, 47]. However, the findings of the present study may be due to the uncontrolled effect of other variables and small sample size. Improvement of clean and adequate water, sanitation and hygiene were identified as evidence-based interventions to prevent low birth weight [6]. Additionally, further study is required to better elucidate the relationship of low birth weight with these variables. As most deliveries in Ethiopia occurred at home [8], these counterintuitive results might be due to differences in the analytical sample and the general population.

As strength, the authors excluded known risk factors of low birth weight to minimize possible confounding. Additionally, the authors selected institution-based cases to minimize selection bias. However, this study may be prone to recall bias because the respondents were asked about their previous obstetric characteristics. Moreover, some confounders may not be controlled due to unmatched selection of study subjects. Generalizability of the results to the general population should be made cautiously, as observation was not made for house and environmental factors.

## Conclusion

This study showed that socio-demographic, maternal/obstetric and environmental characteristics were risk factors for low birth weight in the study areas. Husband educational status, place of ANC visits, previous obstetric complications, maternal weight during pregnancy and gravidity were significantly associated with low birth weight. Nutritional counseling during ANC visits for pregnant mothers and health information about obstetric complications should be advocated. Health professionals should be vigilant in early detection and management of complications during pregnancy. Additionally, efforts should be done to improve living standard and lifestyles of mothers. Community based studies are needed to better address household and environmental factors with observation.

## Supporting information

S1 Table (DOCX)

S1 File (SAV)
