# Supplementary material for: Risk factors for low birth weight in hospitals of North Wello zone, Ethiopia: A case-control study
Source: PLoS One. 2019 Mar 20;14(3):e0213054. doi: 10.1371/journal.pone.0213054 (PMC6426181; doi:10.1371/journal.pone.0213054)
Supplement: S1 Table — (DOCX) [file pone.0213054.s001.docx]

1. Sensitivity (with BMI category instead of weight and height in the model)

| **Hosmer and Lemeshow Test** | | | |
| --- | --- | --- | --- |
| Step | Chi-square | df | Sig. |
| 1 | 9.692 | 8 | .287 |
| 2 | 14.385 | 8 | .072 |
| 3 | 10.596 | 8 | .226 |
| 4 | 12.226 | 8 | .141 |
| 5 | 10.862 | 8 | .210 |
| 6 | 18.800 | 8 | .016 |
| 7 | 7.712 | 8 | .462 |
| 8 | 10.071 | 8 | .260 |
| 9 | 21.674 | 8 | .006 |
| 10 | 20.552 | 8 | .008 |

| **Classification Table^a^** | | | | | |
| --- | --- | --- | --- | --- | --- |
|  | Observed | | Predicted | | |
|  |  |  | lbw | | Percentage Correct |
|  |  |  | nbw | lbw |  |
| Step 1 | lbw | nbw | 153 | 17 | 90.0 |
|  |  | lbw | 36 | 48 | 57.1 |
|  | Overall Percentage | |  |  | 79.1 |
| Step 2 | lbw | nbw | 152 | 18 | 89.4 |
|  |  | lbw | 36 | 48 | 57.1 |
|  | Overall Percentage | |  |  | 78.7 |
| Step 3 | lbw | nbw | 152 | 18 | 89.4 |
|  |  | lbw | 37 | 47 | 56.0 |
|  | Overall Percentage | |  |  | 78.3 |
| Step 4 | lbw | nbw | 153 | 17 | 90.0 |
|  |  | lbw | 36 | 48 | 57.1 |
|  | Overall Percentage | |  |  | 79.1 |
| Step 5 | lbw | nbw | 156 | 14 | 91.8 |
|  |  | lbw | 38 | 46 | 54.8 |
|  | Overall Percentage | |  |  | 79.5 |
| Step 6 | lbw | nbw | 158 | 12 | 92.9 |
|  |  | lbw | 38 | 46 | 54.8 |
|  | Overall Percentage | |  |  | 80.3 |
| Step 7 | lbw | nbw | 158 | 12 | 92.9 |
|  |  | lbw | 38 | 46 | 54.8 |
|  | Overall Percentage | |  |  | 80.3 |
| Step 8 | lbw | nbw | 158 | 12 | 92.9 |
|  |  | lbw | 37 | 47 | 56.0 |
|  | Overall Percentage | |  |  | 80.7 |
| Step 9 | lbw | nbw | 163 | 7 | 95.9 |
|  |  | lbw | 38 | 46 | 54.8 |
|  | Overall Percentage | |  |  | 82.3 |
| Step 10 | lbw | nbw | 154 | 16 | 90.6 |
|  |  | lbw | 38 | 46 | 54.8 |
|  | Overall Percentage | |  |  | **78.7** |
| a. The cut value is .500 | | | | | |

| **Variables in the Equation** | | | | | | | | | |
| --- | --- | --- | --- | --- | --- | --- | --- | --- | --- |
|  | | B | S.E. | Wald | df | Sig. | Exp(B) | 95% C.I.for EXP(B) | |
|  |  |  |  |  |  |  |  | Lower | Upper |
| Step 1^a^ | place_cat(1) | -2.042 | .910 | 5.041 | 1 | .025 | .130 | .022 | .771 |
|  | informed_on_signsof_PCX_at_ANC_visit(1) | -.391 | .472 | .685 | 1 | .408 | .676 | .268 | 1.707 |
|  | have_hx_of_cxn(1) | -1.646 | .517 | 10.136 | 1 | .001 | .193 | .070 | .531 |
|  | BMI_cat |  |  | 3.847 | 2 | .146 |  |  |  |
|  | BMI_cat(1) | 1.535 | .940 | 2.667 | 1 | .102 | 4.642 | .735 | 29.303 |
|  | BMI_cat(2) | .784 | .470 | 2.788 | 1 | .095 | 2.190 | .873 | 5.498 |
|  | group_mat_age |  |  | .309 | 2 | .857 |  |  |  |
|  | group_mat_age(1) | .215 | 1.183 | .033 | 1 | .856 | 1.240 | .122 | 12.596 |
|  | group_mat_age(2) | .481 | 1.164 | .171 | 1 | .679 | 1.618 | .165 | 15.840 |
|  | grav_cat |  |  | 6.327 | 2 | .042 |  |  |  |
|  | grav_cat(1) | -.990 | .396 | 6.250 | 1 | .012 | .372 | .171 | .808 |
|  | grav_cat(2) | -.685 | 1.026 | .446 | 1 | .504 | .504 | .067 | 3.769 |
|  | wk_1st_ANC |  |  | 2.819 | 2 | .244 |  |  |  |
|  | wk_1st_ANC(1) | .112 | .412 | .075 | 1 | .785 | 1.119 | .499 | 2.508 |
|  | wk_1st_ANC(2) | .837 | .521 | 2.584 | 1 | .108 | 2.310 | .832 | 6.413 |
|  | chewing_of_khat(1) | .496 | .761 | .426 | 1 | .514 | 1.643 | .370 | 7.293 |
|  | source_cat(1) | -.291 | .611 | .226 | 1 | .634 | .748 | .226 | 2.475 |
|  | storage_cat(1) | -1.545 | .391 | 15.598 | 1 | .000 | .213 | .099 | .459 |
|  | treating_water(1) | -1.495 | .416 | 12.894 | 1 | .000 | .224 | .099 | .507 |
|  | handwashing_cat(1) | -.852 | .509 | 2.803 | 1 | .094 | .427 | .157 | 1.157 |
|  | separateroom_for_kitchen(1) | .327 | .533 | .377 | 1 | .539 | 1.387 | .488 | 3.939 |
|  | group_water_consump(1) | .355 | .484 | .538 | 1 | .463 | 1.426 | .553 | 3.679 |
|  | windows_cat(1) | .335 | .624 | .288 | 1 | .591 | 1.398 | .412 | 4.749 |
|  | husband_educationalstatus |  |  | 7.093 | 3 | .069 |  |  |  |
|  | husband_educationalstatus(1) | 1.686 | .704 | 5.726 | 1 | .017 | 5.396 | 1.357 | 21.465 |
|  | husband_educationalstatus(2) | .820 | .479 | 2.938 | 1 | .087 | 2.272 | .889 | 5.805 |
|  | husband_educationalstatus(3) | -.120 | .523 | .053 | 1 | .819 | .887 | .319 | 2.470 |
|  | Constant | .917 | 1.390 | .436 | 1 | .509 | 2.502 |  |  |
| Step 2^a^ | place_cat(1) | -1.964 | .893 | 4.833 | 1 | .028 | .140 | .024 | .808 |
|  | informed_on_signsof_PCX_at_ANC_visit(1) | -.388 | .462 | .704 | 1 | .401 | .678 | .274 | 1.679 |
|  | have_hx_of_cxn(1) | -1.620 | .511 | 10.040 | 1 | .002 | .198 | .073 | .539 |
|  | BMI_cat |  |  | 4.157 | 2 | .125 |  |  |  |
|  | BMI_cat(1) | 1.583 | .932 | 2.882 | 1 | .090 | 4.869 | .783 | 30.280 |
|  | BMI_cat(2) | .808 | .466 | 3.010 | 1 | .083 | 2.243 | .900 | 5.588 |
|  | grav_cat |  |  | 6.974 | 2 | .031 |  |  |  |
|  | grav_cat(1) | -.938 | .378 | 6.141 | 1 | .013 | .391 | .186 | .822 |
|  | grav_cat(2) | -1.019 | .564 | 3.261 | 1 | .071 | .361 | .120 | 1.091 |
|  | wk_1st_ANC |  |  | 3.099 | 2 | .212 |  |  |  |
|  | wk_1st_ANC(1) | .109 | .410 | .070 | 1 | .791 | 1.115 | .499 | 2.492 |
|  | wk_1st_ANC(2) | .845 | .503 | 2.821 | 1 | .093 | 2.329 | .868 | 6.245 |
|  | chewing_of_khat(1) | .506 | .756 | .447 | 1 | .504 | 1.658 | .377 | 7.301 |
|  | source_cat(1) | -.246 | .578 | .181 | 1 | .671 | .782 | .252 | 2.430 |
|  | storage_cat(1) | -1.532 | .390 | 15.432 | 1 | .000 | .216 | .101 | .464 |
|  | treating_water(1) | -1.512 | .415 | 13.253 | 1 | .000 | .220 | .098 | .498 |
|  | handwashing_cat(1) | -.807 | .501 | 2.593 | 1 | .107 | .446 | .167 | 1.192 |
|  | separateroom_for_kitchen(1) | .280 | .523 | .288 | 1 | .592 | 1.324 | .475 | 3.689 |
|  | group_water_consump(1) | .388 | .480 | .652 | 1 | .419 | 1.473 | .575 | 3.774 |
|  | windows_cat(1) | .342 | .621 | .304 | 1 | .582 | 1.408 | .417 | 4.753 |
|  | husband_educationalstatus |  |  | 6.924 | 3 | .074 |  |  |  |
|  | husband_educationalstatus(1) | 1.620 | .690 | 5.518 | 1 | .019 | 5.054 | 1.308 | 19.533 |
|  | husband_educationalstatus(2) | .811 | .477 | 2.890 | 1 | .089 | 2.250 | .883 | 5.732 |
|  | husband_educationalstatus(3) | -.135 | .521 | .067 | 1 | .796 | .874 | .315 | 2.425 |
|  | Constant | 1.276 | .965 | 1.749 | 1 | .186 | 3.583 |  |  |
| Step 3^a^ | place_cat(1) | -1.899 | .874 | 4.714 | 1 | .030 | .150 | .027 | .831 |
|  | informed_on_signsof_PCX_at_ANC_visit(1) | -.419 | .457 | .841 | 1 | .359 | .657 | .268 | 1.611 |
|  | have_hx_of_cxn(1) | -1.575 | .498 | 9.986 | 1 | .002 | .207 | .078 | .550 |
|  | BMI_cat |  |  | 4.633 | 2 | .099 |  |  |  |
|  | BMI_cat(1) | 1.648 | .923 | 3.188 | 1 | .074 | 5.197 | .851 | 31.733 |
|  | BMI_cat(2) | .837 | .460 | 3.312 | 1 | .069 | 2.310 | .938 | 5.692 |
|  | grav_cat |  |  | 6.821 | 2 | .033 |  |  |  |
|  | grav_cat(1) | -.909 | .372 | 5.984 | 1 | .014 | .403 | .194 | .835 |
|  | grav_cat(2) | -1.026 | .563 | 3.323 | 1 | .068 | .358 | .119 | 1.080 |
|  | wk_1st_ANC |  |  | 2.898 | 2 | .235 |  |  |  |
|  | wk_1st_ANC(1) | .144 | .401 | .129 | 1 | .719 | 1.155 | .526 | 2.536 |
|  | wk_1st_ANC(2) | .812 | .498 | 2.651 | 1 | .103 | 2.252 | .848 | 5.981 |
|  | chewing_of_khat(1) | .484 | .759 | .407 | 1 | .523 | 1.623 | .367 | 7.182 |
|  | storage_cat(1) | -1.513 | .386 | 15.332 | 1 | .000 | .220 | .103 | .470 |
|  | treating_water(1) | -1.533 | .413 | 13.793 | 1 | .000 | .216 | .096 | .485 |
|  | handwashing_cat(1) | -.794 | .500 | 2.518 | 1 | .113 | .452 | .170 | 1.205 |
|  | separateroom_for_kitchen(1) | .255 | .522 | .238 | 1 | .625 | 1.290 | .464 | 3.590 |
|  | group_water_consump(1) | .366 | .476 | .591 | 1 | .442 | 1.442 | .567 | 3.666 |
|  | windows_cat(1) | .364 | .623 | .341 | 1 | .559 | 1.439 | .425 | 4.877 |
|  | husband_educationalstatus |  |  | 6.761 | 3 | .080 |  |  |  |
|  | husband_educationalstatus(1) | 1.554 | .672 | 5.350 | 1 | .021 | 4.732 | 1.268 | 17.665 |
|  | husband_educationalstatus(2) | .826 | .477 | 3.001 | 1 | .083 | 2.285 | .897 | 5.818 |
|  | husband_educationalstatus(3) | -.094 | .510 | .034 | 1 | .854 | .910 | .335 | 2.475 |
|  | Constant | 1.184 | .935 | 1.602 | 1 | .206 | 3.266 |  |  |
| Step 4^a^ | place_cat(1) | -1.930 | .873 | 4.892 | 1 | .027 | .145 | .026 | .803 |
|  | informed_on_signsof_PCX_at_ANC_visit(1) | -.419 | .457 | .839 | 1 | .360 | .658 | .268 | 1.612 |
|  | have_hx_of_cxn(1) | -1.597 | .495 | 10.421 | 1 | .001 | .203 | .077 | .534 |
|  | BMI_cat |  |  | 4.532 | 2 | .104 |  |  |  |
|  | BMI_cat(1) | 1.612 | .917 | 3.094 | 1 | .079 | 5.014 | .832 | 30.233 |
|  | BMI_cat(2) | .819 | .455 | 3.236 | 1 | .072 | 2.268 | .929 | 5.536 |
|  | grav_cat |  |  | 6.843 | 2 | .033 |  |  |  |
|  | grav_cat(1) | -.915 | .372 | 6.048 | 1 | .014 | .401 | .193 | .831 |
|  | grav_cat(2) | -1.016 | .561 | 3.287 | 1 | .070 | .362 | .121 | 1.086 |
|  | wk_1st_ANC |  |  | 2.909 | 2 | .233 |  |  |  |
|  | wk_1st_ANC(1) | .160 | .400 | .159 | 1 | .690 | 1.173 | .535 | 2.570 |
|  | wk_1st_ANC(2) | .820 | .499 | 2.702 | 1 | .100 | 2.269 | .854 | 6.029 |
|  | chewing_of_khat(1) | .498 | .754 | .436 | 1 | .509 | 1.646 | .375 | 7.221 |
|  | storage_cat(1) | -1.500 | .385 | 15.201 | 1 | .000 | .223 | .105 | .474 |
|  | treating_water(1) | -1.506 | .409 | 13.543 | 1 | .000 | .222 | .099 | .495 |
|  | handwashing_cat(1) | -.772 | .498 | 2.400 | 1 | .121 | .462 | .174 | 1.227 |
|  | group_water_consump(1) | .397 | .472 | .708 | 1 | .400 | 1.487 | .590 | 3.749 |
|  | windows_cat(1) | .483 | .575 | .706 | 1 | .401 | 1.621 | .525 | 5.006 |
|  | husband_educationalstatus |  |  | 8.081 | 3 | .044 |  |  |  |
|  | husband_educationalstatus(1) | 1.648 | .644 | 6.548 | 1 | .011 | 5.195 | 1.471 | 18.353 |
|  | husband_educationalstatus(2) | .875 | .466 | 3.524 | 1 | .060 | 2.400 | .962 | 5.986 |
|  | husband_educationalstatus(3) | -.067 | .507 | .017 | 1 | .895 | .935 | .347 | 2.525 |
|  | Constant | 1.182 | .936 | 1.593 | 1 | .207 | 3.260 |  |  |
| Step 5^a^ | place_cat(1) | -1.950 | .872 | 4.994 | 1 | .025 | .142 | .026 | .787 |
|  | informed_on_signsof_PCX_at_ANC_visit(1) | -.352 | .443 | .629 | 1 | .428 | .704 | .295 | 1.678 |
|  | have_hx_of_cxn(1) | -1.599 | .494 | 10.475 | 1 | .001 | .202 | .077 | .532 |
|  | BMI_cat |  |  | 4.657 | 2 | .097 |  |  |  |
|  | BMI_cat(1) | 1.685 | .905 | 3.466 | 1 | .063 | 5.395 | .915 | 31.812 |
|  | BMI_cat(2) | .795 | .453 | 3.087 | 1 | .079 | 2.215 | .912 | 5.379 |
|  | grav_cat |  |  | 7.137 | 2 | .028 |  |  |  |
|  | grav_cat(1) | -.921 | .371 | 6.163 | 1 | .013 | .398 | .193 | .824 |
|  | grav_cat(2) | -1.066 | .557 | 3.660 | 1 | .056 | .344 | .116 | 1.026 |
|  | wk_1st_ANC |  |  | 2.612 | 2 | .271 |  |  |  |
|  | wk_1st_ANC(1) | .189 | .396 | .228 | 1 | .633 | 1.208 | .556 | 2.623 |
|  | wk_1st_ANC(2) | .779 | .495 | 2.477 | 1 | .116 | 2.180 | .826 | 5.752 |
|  | storage_cat(1) | -1.534 | .381 | 16.211 | 1 | .000 | .216 | .102 | .455 |
|  | treating_water(1) | -1.471 | .404 | 13.231 | 1 | .000 | .230 | .104 | .507 |
|  | handwashing_cat(1) | -.751 | .499 | 2.265 | 1 | .132 | .472 | .178 | 1.255 |
|  | group_water_consump(1) | .423 | .471 | .809 | 1 | .368 | 1.527 | .607 | 3.842 |
|  | windows_cat(1) | .523 | .569 | .843 | 1 | .359 | 1.686 | .553 | 5.148 |
|  | husband_educationalstatus |  |  | 9.005 | 3 | .029 |  |  |  |
|  | husband_educationalstatus(1) | 1.739 | .628 | 7.669 | 1 | .006 | 5.694 | 1.663 | 19.500 |
|  | husband_educationalstatus(2) | .873 | .465 | 3.518 | 1 | .061 | 2.394 | .962 | 5.959 |
|  | husband_educationalstatus(3) | -.067 | .508 | .017 | 1 | .895 | .935 | .346 | 2.531 |
|  | Constant | 1.171 | .936 | 1.564 | 1 | .211 | 3.226 |  |  |
| Step 6^a^ | place_cat(1) | -2.081 | .861 | 5.836 | 1 | .016 | .125 | .023 | .675 |
|  | have_hx_of_cxn(1) | -1.697 | .479 | 12.530 | 1 | .000 | .183 | .072 | .469 |
|  | BMI_cat |  |  | 4.587 | 2 | .101 |  |  |  |
|  | BMI_cat(1) | 1.697 | .908 | 3.492 | 1 | .062 | 5.459 | .920 | 32.384 |
|  | BMI_cat(2) | .775 | .451 | 2.952 | 1 | .086 | 2.171 | .897 | 5.259 |
|  | grav_cat |  |  | 7.708 | 2 | .021 |  |  |  |
|  | grav_cat(1) | -.958 | .368 | 6.788 | 1 | .009 | .383 | .186 | .789 |
|  | grav_cat(2) | -1.073 | .552 | 3.774 | 1 | .052 | .342 | .116 | 1.010 |
|  | wk_1st_ANC |  |  | 2.523 | 2 | .283 |  |  |  |
|  | wk_1st_ANC(1) | .166 | .394 | .178 | 1 | .673 | 1.181 | .545 | 2.557 |
|  | wk_1st_ANC(2) | .758 | .493 | 2.359 | 1 | .125 | 2.134 | .811 | 5.611 |
|  | storage_cat(1) | -1.497 | .376 | 15.859 | 1 | .000 | .224 | .107 | .468 |
|  | treating_water(1) | -1.411 | .397 | 12.634 | 1 | .000 | .244 | .112 | .531 |
|  | handwashing_cat(1) | -.770 | .494 | 2.433 | 1 | .119 | .463 | .176 | 1.218 |
|  | group_water_consump(1) | .352 | .462 | .580 | 1 | .446 | 1.422 | .575 | 3.516 |
|  | windows_cat(1) | .402 | .546 | .542 | 1 | .461 | 1.495 | .513 | 4.358 |
|  | husband_educationalstatus |  |  | 9.363 | 3 | .025 |  |  |  |
|  | husband_educationalstatus(1) | 1.756 | .624 | 7.927 | 1 | .005 | 5.789 | 1.705 | 19.657 |
|  | husband_educationalstatus(2) | .856 | .461 | 3.454 | 1 | .063 | 2.354 | .954 | 5.806 |
|  | husband_educationalstatus(3) | -.098 | .507 | .038 | 1 | .846 | .906 | .336 | 2.448 |
|  | Constant | 1.271 | .925 | 1.891 | 1 | .169 | 3.566 |  |  |
| Step 7^a^ | place_cat(1) | -2.125 | .860 | 6.100 | 1 | .014 | .119 | .022 | .645 |
|  | have_hx_of_cxn(1) | -1.685 | .478 | 12.431 | 1 | .000 | .185 | .073 | .473 |
|  | BMI_cat |  |  | 4.612 | 2 | .100 |  |  |  |
|  | BMI_cat(1) | 1.712 | .907 | 3.563 | 1 | .059 | 5.542 | .936 | 32.801 |
|  | BMI_cat(2) | .773 | .451 | 2.932 | 1 | .087 | 2.166 | .894 | 5.248 |
|  | grav_cat |  |  | 7.722 | 2 | .021 |  |  |  |
|  | grav_cat(1) | -.966 | .368 | 6.906 | 1 | .009 | .381 | .185 | .782 |
|  | grav_cat(2) | -1.055 | .552 | 3.656 | 1 | .056 | .348 | .118 | 1.027 |
|  | wk_1st_ANC |  |  | 2.299 | 2 | .317 |  |  |  |
|  | wk_1st_ANC(1) | .149 | .393 | .143 | 1 | .705 | 1.160 | .537 | 2.508 |
|  | wk_1st_ANC(2) | .709 | .485 | 2.135 | 1 | .144 | 2.033 | .785 | 5.263 |
|  | storage_cat(1) | -1.506 | .376 | 16.042 | 1 | .000 | .222 | .106 | .463 |
|  | treating_water(1) | -1.345 | .386 | 12.147 | 1 | .000 | .261 | .122 | .555 |
|  | handwashing_cat(1) | -.676 | .472 | 2.049 | 1 | .152 | .508 | .201 | 1.284 |
|  | group_water_consump(1) | .417 | .451 | .855 | 1 | .355 | 1.518 | .627 | 3.675 |
|  | husband_educationalstatus |  |  | 10.979 | 3 | .012 |  |  |  |
|  | husband_educationalstatus(1) | 1.815 | .616 | 8.688 | 1 | .003 | 6.144 | 1.837 | 20.546 |
|  | husband_educationalstatus(2) | .949 | .443 | 4.588 | 1 | .032 | 2.584 | 1.084 | 6.158 |
|  | husband_educationalstatus(3) | -.112 | .506 | .049 | 1 | .824 | .894 | .331 | 2.410 |
|  | Constant | 1.207 | .921 | 1.718 | 1 | .190 | 3.343 |  |  |
| Step 8^a^ | place_cat(1) | -2.261 | .846 | 7.145 | 1 | .008 | .104 | .020 | .547 |
|  | have_hx_of_cxn(1) | -1.753 | .474 | 13.677 | 1 | .000 | .173 | .068 | .439 |
|  | BMI_cat |  |  | 4.375 | 2 | .112 |  |  |  |
|  | BMI_cat(1) | 1.703 | .915 | 3.464 | 1 | .063 | 5.488 | .914 | 32.963 |
|  | BMI_cat(2) | .732 | .449 | 2.661 | 1 | .103 | 2.079 | .863 | 5.011 |
|  | grav_cat |  |  | 8.373 | 2 | .015 |  |  |  |
|  | grav_cat(1) | -1.003 | .365 | 7.569 | 1 | .006 | .367 | .180 | .749 |
|  | grav_cat(2) | -1.081 | .556 | 3.782 | 1 | .052 | .339 | .114 | 1.008 |
|  | wk_1st_ANC |  |  | 2.362 | 2 | .307 |  |  |  |
|  | wk_1st_ANC(1) | .165 | .391 | .179 | 1 | .672 | 1.180 | .548 | 2.540 |
|  | wk_1st_ANC(2) | .722 | .484 | 2.226 | 1 | .136 | 2.059 | .797 | 5.320 |
|  | storage_cat(1) | -1.465 | .374 | 15.357 | 1 | .000 | .231 | .111 | .481 |
|  | treating_water(1) | -1.418 | .379 | 14.007 | 1 | .000 | .242 | .115 | .509 |
|  | handwashing_cat(1) | -.693 | .472 | 2.154 | 1 | .142 | .500 | .198 | 1.262 |
|  | husband_educationalstatus |  |  | 11.662 | 3 | .009 |  |  |  |
|  | husband_educationalstatus(1) | 1.846 | .619 | 8.878 | 1 | .003 | 6.332 | 1.881 | 21.319 |
|  | husband_educationalstatus(2) | .978 | .442 | 4.900 | 1 | .027 | 2.659 | 1.119 | 6.322 |
|  | husband_educationalstatus(3) | -.154 | .502 | .095 | 1 | .758 | .857 | .321 | 2.291 |
|  | Constant | 1.679 | .769 | 4.760 | 1 | .029 | 5.358 |  |  |
| Step 9^a^ | place_cat(1) | -2.164 | .827 | 6.851 | 1 | .009 | .115 | .023 | .581 |
|  | have_hx_of_cxn(1) | -1.594 | .453 | 12.358 | 1 | .000 | .203 | .084 | .494 |
|  | BMI_cat |  |  | 4.902 | 2 | .086 |  |  |  |
|  | BMI_cat(1) | 1.894 | .936 | 4.100 | 1 | .043 | 6.648 | 1.063 | 41.593 |
|  | BMI_cat(2) | .712 | .442 | 2.597 | 1 | .107 | 2.039 | .857 | 4.849 |
|  | grav_cat |  |  | 8.287 | 2 | .016 |  |  |  |
|  | grav_cat(1) | -.973 | .356 | 7.488 | 1 | .006 | .378 | .188 | .759 |
|  | grav_cat(2) | -1.046 | .553 | 3.576 | 1 | .059 | .351 | .119 | 1.039 |
|  | storage_cat(1) | -1.370 | .362 | 14.354 | 1 | .000 | .254 | .125 | .516 |
|  | treating_water(1) | -1.398 | .371 | 14.224 | 1 | .000 | .247 | .119 | .511 |
|  | handwashing_cat(1) | -.630 | .469 | 1.805 | 1 | .179 | .532 | .212 | 1.335 |
|  | husband_educationalstatus |  |  | 13.429 | 3 | .004 |  |  |  |
|  | husband_educationalstatus(1) | 1.977 | .619 | 10.215 | 1 | .001 | 7.222 | 2.148 | 24.280 |
|  | husband_educationalstatus(2) | 1.044 | .443 | 5.562 | 1 | .018 | 2.840 | 1.193 | 6.761 |
|  | husband_educationalstatus(3) | -.167 | .492 | .114 | 1 | .735 | .847 | .323 | 2.222 |
|  | Constant | 1.669 | .703 | 5.639 | 1 | .018 | 5.309 |  |  |
| **Step 10^a^** | place_cat(1) | -2.043 | .826 | 6.112 | 1 | .013 | .130 | .026 | .655 |
|  | have_hx_of_cxn(1) | -1.624 | .454 | 12.818 | 1 | .000 | .197 | .081 | .479 |
|  | BMI_cat |  |  | 5.854 | 2 | .054 |  |  |  |
|  | BMI_cat(1) | 2.042 | .926 | 4.861 | 1 | .027 | 7.702 | 1.254 | 47.292 |
|  | BMI_cat(2) | .770 | .436 | 3.115 | 1 | .078 | 2.159 | .918 | 5.077 |
|  | grav_cat |  |  | 8.844 | 2 | .012 |  |  |  |
|  | grav_cat(1) | -1.017 | .353 | 8.284 | 1 | .004 | .362 | .181 | .723 |
|  | grav_cat(2) | -1.002 | .545 | 3.383 | 1 | .066 | .367 | .126 | 1.068 |
|  | storage_cat(1) | -1.352 | .360 | 14.091 | 1 | .000 | .259 | .128 | .524 |
|  | treating_water(1) | -1.463 | .368 | 15.848 | 1 | .000 | .232 | .113 | .476 |
|  | husband_educationalstatus |  |  | 12.500 | 3 | .006 |  |  |  |
|  | husband_educationalstatus(1) | 1.617 | .546 | 8.784 | 1 | .003 | 5.040 | 1.730 | 14.687 |
|  | husband_educationalstatus(2) | .813 | .406 | 4.008 | 1 | .045 | 2.254 | 1.017 | 4.996 |
|  | husband_educationalstatus(3) | -.191 | .490 | .152 | 1 | .696 | .826 | .316 | 2.160 |
|  | Constant | 1.650 | .698 | 5.593 | 1 | .018 | 5.208 |  |  |
| a. Variable(s) entered on step 1: place_cat, informed_on_signsof_PCX_at_ANC_visit, have_hx_of_cxn, BMI_cat, group_mat_age, grav_cat, wk_1st_ANC, chewing_of_khat, source_cat, storage_cat, treating_water, handwashing_cat, separateroom_for_kitchen, group_water_consump, windows_cat, husband_educationalstatus. | | | | | | | | | |

1. Sensitivity (after removing potential intermediates)

| **Classification Table^a^** | | | | | |
| --- | --- | --- | --- | --- | --- |
|  | Observed | | Predicted | | |
|  |  |  | lbw | | Percentage Correct |
|  |  |  | nbw | lbw |  |
| Step 1 | lbw | nbw | 155 | 15 | 91.2 |
|  |  | lbw | 40 | 44 | 52.4 |
|  | Overall Percentage | |  |  | 78.3 |
| Step 2 | lbw | nbw | 155 | 15 | 91.2 |
|  |  | lbw | 42 | 42 | 50.0 |
|  | Overall Percentage | |  |  | 77.6 |
| Step 3 | lbw | nbw | 156 | 14 | 91.8 |
|  |  | lbw | 43 | 41 | 48.8 |
|  | Overall Percentage | |  |  | 77.6 |
| Step 4 | lbw | nbw | 153 | 17 | 90.0 |
|  |  | lbw | 39 | 45 | 53.6 |
|  | Overall Percentage | |  |  | 78.0 |
| Step 5 | lbw | nbw | 160 | 10 | 94.1 |
|  |  | lbw | 41 | 43 | 51.2 |
|  | Overall Percentage | |  |  | 79.9 |
| Step 6 | lbw | nbw | 161 | 9 | 94.7 |
|  |  | lbw | 42 | 42 | 50.0 |
|  | Overall Percentage | |  |  | 79.9 |
| Step 7 | lbw | nbw | 161 | 9 | 94.7 |
|  |  | lbw | 42 | 42 | 50.0 |
|  | Overall Percentage | |  |  | 79.9 |
| Step 8 | lbw | nbw | 161 | 9 | 94.7 |
|  |  | lbw | 39 | 45 | 53.6 |
|  | Overall Percentage | |  |  | 81.1 |
| a. The cut value is .500 | | | | | |

| **Variables in the Equation** | | | | | | | | | |
| --- | --- | --- | --- | --- | --- | --- | --- | --- | --- |
|  | | B | S.E. | Wald | df | Sig. | Exp(B) | 95% C.I.for EXP(B) | |
|  |  |  |  |  |  |  |  | Lower | Upper |
| Step 1^a^ | place_cat(1) | -2.278 | .878 | 6.739 | 1 | .009 | .102 | .018 | .572 |
|  | have_hx_of_cxn(1) | 1.976 | .493 | 16.080 | 1 | .000 | 7.215 | 2.746 | 18.953 |
|  | new_wt_cat(1) | 1.648 | .557 | 8.739 | 1 | .003 | 5.196 | 1.742 | 15.493 |
|  | mat_ht_cat(1) | -.847 | .612 | 1.914 | 1 | .166 | .429 | .129 | 1.423 |
|  | group_mat_age |  |  | .819 | 2 | .664 |  |  |  |
|  | group_mat_age(1) | .231 | 1.144 | .041 | 1 | .840 | 1.259 | .134 | 11.853 |
|  | group_mat_age(2) | .681 | 1.104 | .380 | 1 | .537 | 1.975 | .227 | 17.176 |
|  | grav_cat |  |  | 9.028 | 2 | .011 |  |  |  |
|  | grav_cat(1) | -1.159 | .386 | 9.027 | 1 | .003 | .314 | .147 | .668 |
|  | grav_cat(2) | -.445 | .976 | .208 | 1 | .648 | .641 | .095 | 4.340 |
|  | wk_1st_ANC |  |  | 2.009 | 2 | .366 |  |  |  |
|  | wk_1st_ANC(1) | -.028 | .408 | .005 | 1 | .945 | .972 | .437 | 2.165 |
|  | wk_1st_ANC(2) | .645 | .522 | 1.531 | 1 | .216 | 1.907 | .686 | 5.299 |
|  | chewing_of_khat(1) | .183 | .672 | .074 | 1 | .786 | 1.200 | .321 | 4.485 |
|  | source_cat(1) | -.761 | .599 | 1.618 | 1 | .203 | .467 | .145 | 1.509 |
|  | storage_cat(1) | -1.626 | .397 | 16.746 | 1 | .000 | .197 | .090 | .429 |
|  | treating_water(1) | -1.506 | .415 | 13.187 | 1 | .000 | .222 | .098 | .500 |
|  | separateroom_for_kitchen(1) | .459 | .542 | .717 | 1 | .397 | 1.582 | .547 | 4.576 |
|  | windows_cat(1) | -.068 | .602 | .013 | 1 | .910 | .934 | .287 | 3.038 |
|  | husband_educationalstatus |  |  | 5.225 | 3 | .156 |  |  |  |
|  | husband_educationalstatus(1) | 1.349 | .648 | 4.334 | 1 | .037 | 3.854 | 1.082 | 13.723 |
|  | husband_educationalstatus(2) | .457 | .467 | .955 | 1 | .328 | 1.579 | .632 | 3.944 |
|  | husband_educationalstatus(3) | -.243 | .520 | .219 | 1 | .640 | .784 | .283 | 2.171 |
|  | Constant | .092 | 1.211 | .006 | 1 | .940 | 1.096 |  |  |
| Step 2^a^ | place_cat(1) | -2.272 | .876 | 6.731 | 1 | .009 | .103 | .019 | .574 |
|  | have_hx_of_cxn(1) | 1.976 | .493 | 16.090 | 1 | .000 | 7.215 | 2.747 | 18.949 |
|  | new_wt_cat(1) | 1.641 | .554 | 8.780 | 1 | .003 | 5.159 | 1.743 | 15.272 |
|  | mat_ht_cat(1) | -.853 | .610 | 1.957 | 1 | .162 | .426 | .129 | 1.408 |
|  | group_mat_age |  |  | .807 | 2 | .668 |  |  |  |
|  | group_mat_age(1) | .239 | 1.141 | .044 | 1 | .834 | 1.270 | .136 | 11.873 |
|  | group_mat_age(2) | .681 | 1.103 | .381 | 1 | .537 | 1.976 | .227 | 17.171 |
|  | grav_cat |  |  | 9.022 | 2 | .011 |  |  |  |
|  | grav_cat(1) | -1.157 | .385 | 9.021 | 1 | .003 | .315 | .148 | .669 |
|  | grav_cat(2) | -.447 | .976 | .210 | 1 | .647 | .639 | .094 | 4.327 |
|  | wk_1st_ANC |  |  | 2.033 | 2 | .362 |  |  |  |
|  | wk_1st_ANC(1) | -.024 | .407 | .003 | 1 | .953 | .976 | .440 | 2.167 |
|  | wk_1st_ANC(2) | .651 | .519 | 1.572 | 1 | .210 | 1.918 | .693 | 5.306 |
|  | chewing_of_khat(1) | .169 | .662 | .065 | 1 | .798 | 1.185 | .324 | 4.337 |
|  | source_cat(1) | -.754 | .596 | 1.604 | 1 | .205 | .470 | .146 | 1.512 |
|  | storage_cat(1) | -1.623 | .396 | 16.766 | 1 | .000 | .197 | .091 | .429 |
|  | treating_water(1) | -1.513 | .411 | 13.568 | 1 | .000 | .220 | .098 | .493 |
|  | separateroom_for_kitchen(1) | .430 | .481 | .800 | 1 | .371 | 1.538 | .599 | 3.951 |
|  | husband_educationalstatus |  |  | 5.240 | 3 | .155 |  |  |  |
|  | husband_educationalstatus(1) | 1.345 | .647 | 4.325 | 1 | .038 | 3.839 | 1.081 | 13.640 |
|  | husband_educationalstatus(2) | .448 | .461 | .943 | 1 | .331 | 1.565 | .634 | 3.862 |
|  | husband_educationalstatus(3) | -.239 | .518 | .213 | 1 | .645 | .788 | .285 | 2.174 |
|  | Constant | .090 | 1.210 | .006 | 1 | .941 | 1.094 |  |  |
| Step 3^a^ | place_cat(1) | -2.280 | .875 | 6.786 | 1 | .009 | .102 | .018 | .569 |
|  | have_hx_of_cxn(1) | 1.975 | .493 | 16.032 | 1 | .000 | 7.208 | 2.741 | 18.955 |
|  | new_wt_cat(1) | 1.648 | .553 | 8.888 | 1 | .003 | 5.194 | 1.758 | 15.343 |
|  | mat_ht_cat(1) | -.868 | .608 | 2.039 | 1 | .153 | .420 | .128 | 1.382 |
|  | group_mat_age |  |  | .832 | 2 | .660 |  |  |  |
|  | group_mat_age(1) | .234 | 1.141 | .042 | 1 | .838 | 1.263 | .135 | 11.814 |
|  | group_mat_age(2) | .685 | 1.103 | .385 | 1 | .535 | 1.983 | .228 | 17.222 |
|  | grav_cat |  |  | 9.008 | 2 | .011 |  |  |  |
|  | grav_cat(1) | -1.155 | .385 | 9.007 | 1 | .003 | .315 | .148 | .670 |
|  | grav_cat(2) | -.467 | .972 | .231 | 1 | .631 | .627 | .093 | 4.209 |
|  | wk_1st_ANC |  |  | 1.965 | 2 | .374 |  |  |  |
|  | wk_1st_ANC(1) | -.010 | .403 | .001 | 1 | .980 | .990 | .450 | 2.179 |
|  | wk_1st_ANC(2) | .642 | .519 | 1.531 | 1 | .216 | 1.900 | .687 | 5.250 |
|  | source_cat(1) | -.741 | .593 | 1.562 | 1 | .211 | .477 | .149 | 1.524 |
|  | storage_cat(1) | -1.643 | .389 | 17.812 | 1 | .000 | .193 | .090 | .415 |
|  | treating_water(1) | -1.509 | .410 | 13.529 | 1 | .000 | .221 | .099 | .494 |
|  | separateroom_for_kitchen(1) | .448 | .476 | .886 | 1 | .347 | 1.565 | .616 | 3.981 |
|  | husband_educationalstatus |  |  | 5.548 | 3 | .136 |  |  |  |
|  | husband_educationalstatus(1) | 1.373 | .637 | 4.642 | 1 | .031 | 3.949 | 1.132 | 13.774 |
|  | husband_educationalstatus(2) | .447 | .461 | .942 | 1 | .332 | 1.564 | .634 | 3.856 |
|  | husband_educationalstatus(3) | -.238 | .518 | .212 | 1 | .645 | .788 | .285 | 2.175 |
|  | Constant | .094 | 1.210 | .006 | 1 | .938 | 1.099 |  |  |
| Step 4^a^ | place_cat(1) | -2.189 | .863 | 6.430 | 1 | .011 | .112 | .021 | .608 |
|  | have_hx_of_cxn(1) | 1.945 | .486 | 16.032 | 1 | .000 | 6.997 | 2.700 | 18.134 |
|  | new_wt_cat(1) | 1.607 | .551 | 8.489 | 1 | .004 | 4.986 | 1.692 | 14.694 |
|  | mat_ht_cat(1) | -.878 | .610 | 2.074 | 1 | .150 | .416 | .126 | 1.373 |
|  | grav_cat |  |  | 8.658 | 2 | .013 |  |  |  |
|  | grav_cat(1) | -1.069 | .369 | 8.393 | 1 | .004 | .343 | .166 | .708 |
|  | grav_cat(2) | -.936 | .583 | 2.580 | 1 | .108 | .392 | .125 | 1.229 |
|  | wk_1st_ANC |  |  | 2.299 | 2 | .317 |  |  |  |
|  | wk_1st_ANC(1) | -.018 | .402 | .002 | 1 | .965 | .983 | .446 | 2.162 |
|  | wk_1st_ANC(2) | .668 | .503 | 1.763 | 1 | .184 | 1.950 | .728 | 5.227 |
|  | source_cat(1) | -.682 | .566 | 1.449 | 1 | .229 | .506 | .167 | 1.535 |
|  | storage_cat(1) | -1.624 | .389 | 17.458 | 1 | .000 | .197 | .092 | .422 |
|  | treating_water(1) | -1.531 | .410 | 13.942 | 1 | .000 | .216 | .097 | .483 |
|  | separateroom_for_kitchen(1) | .403 | .476 | .715 | 1 | .398 | 1.496 | .588 | 3.803 |
|  | husband_educationalstatus |  |  | 5.386 | 3 | .146 |  |  |  |
|  | husband_educationalstatus(1) | 1.298 | .624 | 4.323 | 1 | .038 | 3.662 | 1.077 | 12.452 |
|  | husband_educationalstatus(2) | .460 | .460 | .999 | 1 | .318 | 1.584 | .643 | 3.905 |
|  | husband_educationalstatus(3) | -.267 | .514 | .270 | 1 | .603 | .766 | .280 | 2.097 |
|  | Constant | .706 | .523 | 1.820 | 1 | .177 | 2.025 |  |  |
| Step 5^a^ | place_cat(1) | -2.263 | .858 | 6.966 | 1 | .008 | .104 | .019 | .558 |
|  | have_hx_of_cxn(1) | 1.957 | .482 | 16.509 | 1 | .000 | 7.075 | 2.753 | 18.180 |
|  | new_wt_cat(1) | 1.550 | .547 | 8.042 | 1 | .005 | 4.711 | 1.614 | 13.749 |
|  | mat_ht_cat(1) | -.887 | .612 | 2.099 | 1 | .147 | .412 | .124 | 1.367 |
|  | grav_cat |  |  | 8.636 | 2 | .013 |  |  |  |
|  | grav_cat(1) | -1.066 | .369 | 8.347 | 1 | .004 | .344 | .167 | .710 |
|  | grav_cat(2) | -.950 | .581 | 2.676 | 1 | .102 | .387 | .124 | 1.207 |
|  | wk_1st_ANC |  |  | 2.102 | 2 | .350 |  |  |  |
|  | wk_1st_ANC(1) | .021 | .398 | .003 | 1 | .959 | 1.021 | .467 | 2.229 |
|  | wk_1st_ANC(2) | .659 | .501 | 1.734 | 1 | .188 | 1.933 | .725 | 5.158 |
|  | source_cat(1) | -.575 | .553 | 1.082 | 1 | .298 | .563 | .190 | 1.663 |
|  | storage_cat(1) | -1.589 | .385 | 17.056 | 1 | .000 | .204 | .096 | .434 |
|  | treating_water(1) | -1.469 | .403 | 13.280 | 1 | .000 | .230 | .104 | .507 |
|  | husband_educationalstatus |  |  | 8.625 | 3 | .035 |  |  |  |
|  | husband_educationalstatus(1) | 1.499 | .581 | 6.668 | 1 | .010 | 4.479 | 1.435 | 13.977 |
|  | husband_educationalstatus(2) | .625 | .418 | 2.241 | 1 | .134 | 1.869 | .824 | 4.239 |
|  | husband_educationalstatus(3) | -.228 | .510 | .201 | 1 | .654 | .796 | .293 | 2.162 |
|  | Constant | .667 | .522 | 1.633 | 1 | .201 | 1.948 |  |  |
| Step 6^a^ | place_cat(1) | -2.144 | .839 | 6.526 | 1 | .011 | .117 | .023 | .607 |
|  | have_hx_of_cxn(1) | 1.815 | .459 | 15.650 | 1 | .000 | 6.140 | 2.499 | 15.090 |
|  | new_wt_cat(1) | 1.662 | .544 | 9.342 | 1 | .002 | 5.267 | 1.815 | 15.287 |
|  | mat_ht_cat(1) | -.715 | .604 | 1.402 | 1 | .236 | .489 | .150 | 1.597 |
|  | grav_cat |  |  | 8.260 | 2 | .016 |  |  |  |
|  | grav_cat(1) | -1.020 | .360 | 8.023 | 1 | .005 | .360 | .178 | .730 |
|  | grav_cat(2) | -.850 | .569 | 2.229 | 1 | .135 | .427 | .140 | 1.304 |
|  | source_cat(1) | -.440 | .526 | .700 | 1 | .403 | .644 | .230 | 1.806 |
|  | storage_cat(1) | -1.478 | .369 | 16.041 | 1 | .000 | .228 | .111 | .470 |
|  | treating_water(1) | -1.375 | .389 | 12.502 | 1 | .000 | .253 | .118 | .542 |
|  | husband_educationalstatus |  |  | 10.089 | 3 | .018 |  |  |  |
|  | husband_educationalstatus(1) | 1.577 | .565 | 7.793 | 1 | .005 | 4.841 | 1.600 | 14.648 |
|  | husband_educationalstatus(2) | .663 | .419 | 2.504 | 1 | .114 | 1.941 | .854 | 4.416 |
|  | husband_educationalstatus(3) | -.210 | .501 | .175 | 1 | .676 | .811 | .304 | 2.167 |
|  | Constant | .633 | .412 | 2.357 | 1 | .125 | 1.883 |  |  |
| Step 7^a^ | place_cat(1) | -2.071 | .829 | 6.237 | 1 | .013 | .126 | .025 | .640 |
|  | have_hx_of_cxn(1) | 1.747 | .451 | 15.022 | 1 | .000 | 5.739 | 2.372 | 13.885 |
|  | new_wt_cat(1) | 1.544 | .519 | 8.856 | 1 | .003 | 4.683 | 1.694 | 12.945 |
|  | mat_ht_cat(1) | -.764 | .599 | 1.626 | 1 | .202 | .466 | .144 | 1.507 |
|  | grav_cat |  |  | 7.954 | 2 | .019 |  |  |  |
|  | grav_cat(1) | -.986 | .357 | 7.627 | 1 | .006 | .373 | .185 | .751 |
|  | grav_cat(2) | -.901 | .563 | 2.566 | 1 | .109 | .406 | .135 | 1.223 |
|  | storage_cat(1) | -1.454 | .366 | 15.818 | 1 | .000 | .234 | .114 | .478 |
|  | treating_water(1) | -1.426 | .384 | 13.796 | 1 | .000 | .240 | .113 | .510 |
|  | husband_educationalstatus |  |  | 9.474 | 3 | .024 |  |  |  |
|  | husband_educationalstatus(1) | 1.417 | .530 | 7.146 | 1 | .008 | 4.126 | 1.460 | 11.662 |
|  | husband_educationalstatus(2) | .676 | .420 | 2.593 | 1 | .107 | 1.966 | .863 | 4.477 |
|  | husband_educationalstatus(3) | -.162 | .495 | .106 | 1 | .744 | .851 | .322 | 2.247 |
|  | Constant | .614 | .410 | 2.243 | 1 | .134 | 1.847 |  |  |
| Step 8^a^ | place_cat(1) | -2.034 | .829 | 6.013 | 1 | .014 | .131 | .026 | .665 |
|  | have_hx_of_cxn(1) | 1.741 | .445 | 15.333 | 1 | .000 | 5.702 | 2.386 | 13.630 |
|  | new_wt_cat(1) | 1.397 | .503 | 7.702 | 1 | .006 | 4.044 | 1.507 | 10.846 |
|  | grav_cat |  |  | 8.244 | 2 | .016 |  |  |  |
|  | grav_cat(1) | -1.014 | .356 | 8.124 | 1 | .004 | .363 | .181 | .728 |
|  | grav_cat(2) | -.806 | .551 | 2.139 | 1 | .144 | .447 | .152 | 1.315 |
|  | storage_cat(1) | -1.374 | .355 | 14.982 | 1 | .000 | .253 | .126 | .507 |
|  | treating_water(1) | -1.295 | .369 | 12.316 | 1 | .000 | .274 | .133 | .565 |
|  | husband_educationalstatus |  |  | 9.190 | 3 | .027 |  |  |  |
|  | husband_educationalstatus(1) | 1.409 | .530 | 7.064 | 1 | .008 | 4.093 | 1.448 | 11.571 |
|  | husband_educationalstatus(2) | .616 | .418 | 2.172 | 1 | .141 | 1.851 | .816 | 4.196 |
|  | husband_educationalstatus(3) | -.181 | .490 | .136 | 1 | .712 | .835 | .320 | 2.180 |
|  | Constant | .479 | .394 | 1.476 | 1 | .224 | 1.614 |  |  |
| a. Variable(s) entered on step 1: place_cat, have_hx_of_cxn, new_wt_cat, mat_ht_cat, group_mat_age, grav_cat, wk_1st_ANC, chewing_of_khat, source_cat, storage_cat, treating_water, separateroom_for_kitchen, windows_cat, husband_educationalstatus. | | | | | | | | | |
